# Supplementary material for: Identification of efficient prokaryotic cell-penetrating peptides with applications in bacterial biotechnology
Source: Commun Biol. 2021 Feb 15;4:205. doi: 10.1038/s42003-021-01726-w (PMC7884711; doi:10.1038/s42003-021-01726-w)
Supplement: Supplementary file 2 — Supplementary Information [file 42003_2021_1726_MOESM2_ESM.pdf]

Supplementary Information

Identification of efficient prokaryotic cell-penetrating peptides with applications in bacterial biotechnology

Hyang-Mi Lee, Jun Ren, Kha Mong Tran, Byeong-Min Jeon, Won-Ung Park, Hyunjoo Kim, Kyung Eun Lee, Yuna Oh, Myungback Choi, Dae-Sung Kim, and Dokyun Na

List of Supplementary Figures, Tables, and Data

Supplementary Figure 1. Growth curves of *E. coli* cells treated with CPPs to measure cytotoxicity

Supplementary Figure 2. Comparison of the features of CPPs with different cytotoxicities

Supplementary Figure 3. Feature selection and cross-validation result

Supplementary Figure 4. Effects of CPP incubation time on GFP delivery efficiency

Supplementary Figure 5. Activity assay of CPP-conjugated I-SceI

Supplementary Figure 6. Activity assay of CPP-conjugated Cre recombinase

Supplementary Table 1. Determination of the optimal Ni^{++} concentration for the selection of plasmid-removed *E. coli* cells

Supplementary Table 2. Determination of the optimal Ni^{++} concentration for the selection of marker gene-excised *Methylomoas sp*. DH-1 cells

Supplementary Data 1. All datasets used to draw main graphs in this study.

Supplementary Data 2. List of CPPs used in this study.

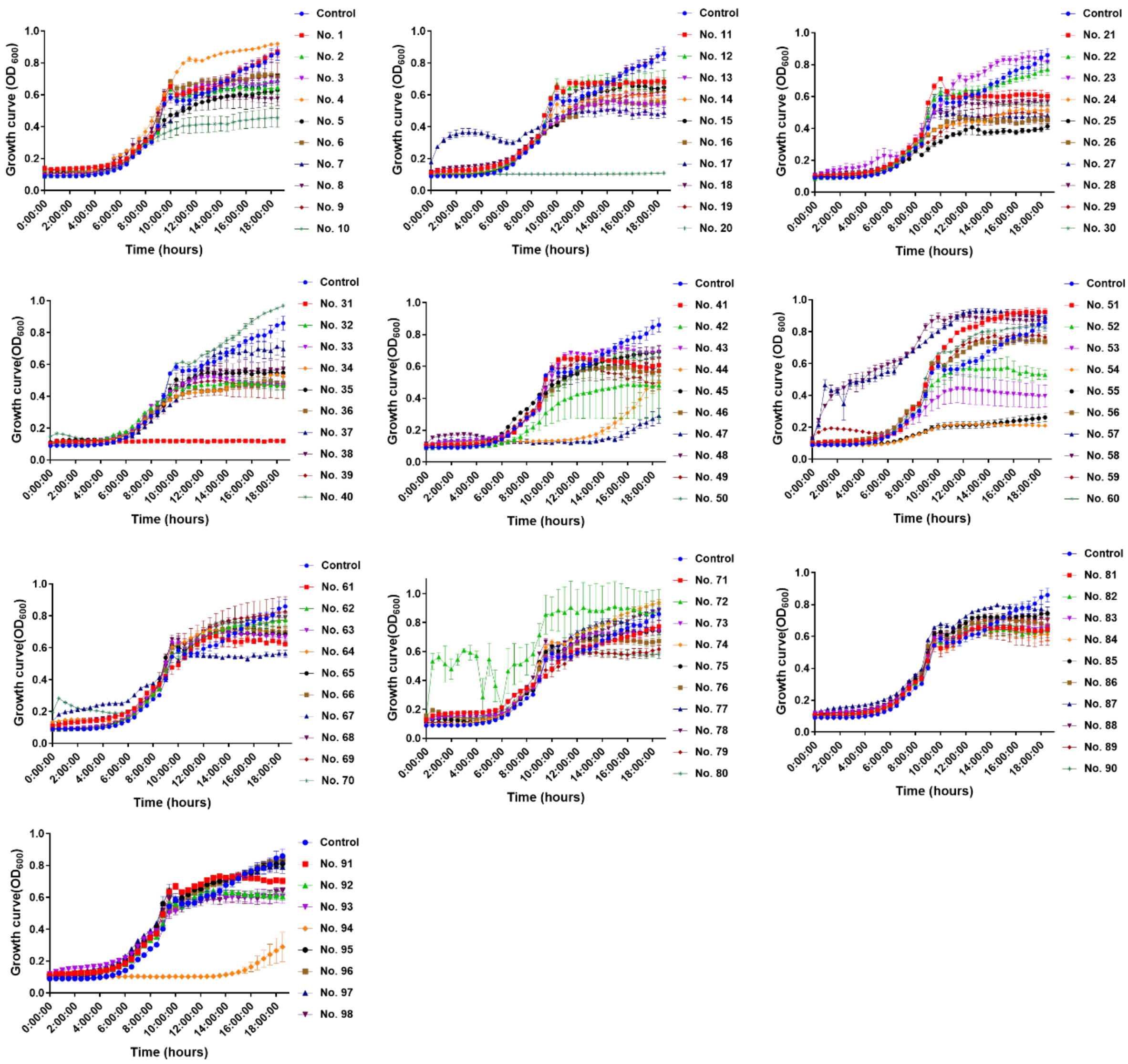

Supplementary Figure 1. Growth curves of *E. coli* cells treated with CPPs to measure cytotoxicity

Control: the growth curve of *E. coli* cells that were not treated with CPPs. **Others**: Growth curves of *E. coli* cells treated with the designated CPPs.

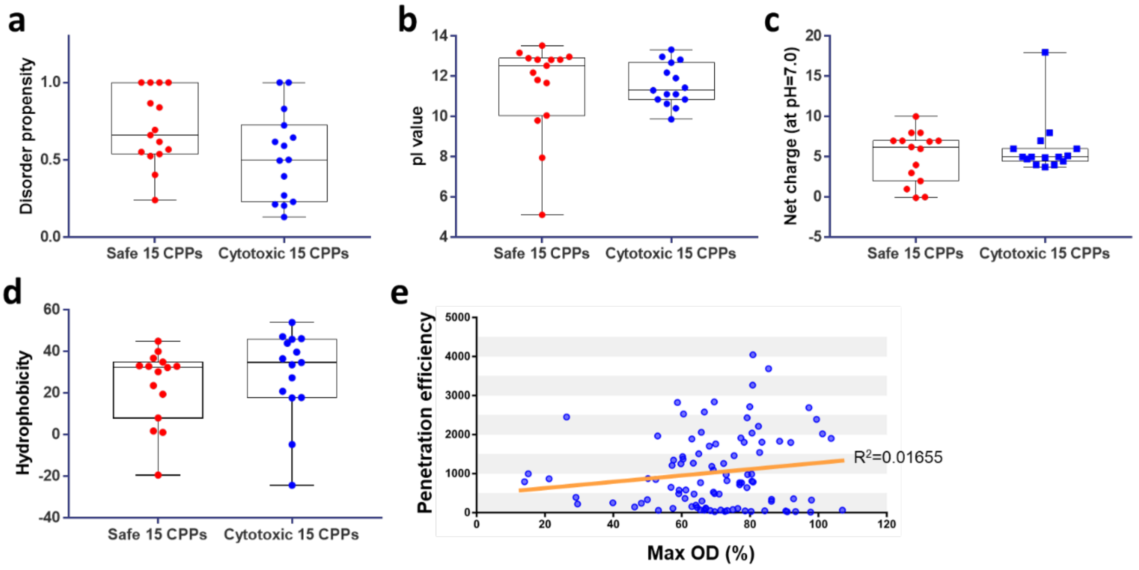

Supplementary Figure 2. Comparison of the features of CPPs with different cytotoxicities

a-d The four sequence features related to penetration efficiency (Fig. 3d, e) were also calculated between the 15 safest CPPs and the 15 most cytotoxic CPPs. The calculated *p*-values (*t*-test) of the four properties were >0.05. Thus, there were no significant differences between the properties of CPPs with different toxicities. **e** The linear correlation between penetration efficiency and max OD (%). Max OD (%) denotes the (max OD of cells treated with CPP)/(max OD of control cells)×100. There was no significant correlation between penetration efficiency and max OD.

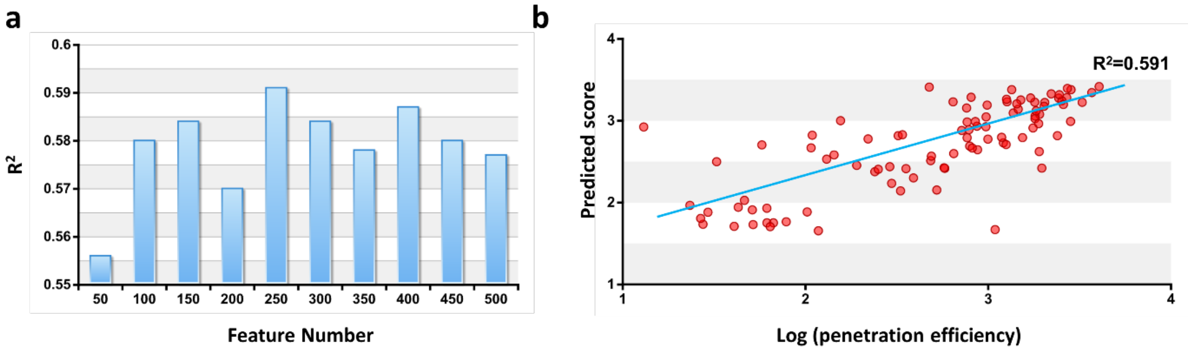

Supplementary Figure 3. Feature selection and cross-validation result

a The 505 features used for the random forest model ranked by their Pearson's correlation coefficients with log (penetration efficiency). The top *n* features were used to build a prediction model, and their R^2 scores are shown. **b** The predicted scores of CPPs resulting from 10-fold cross-validation using 250 features are shown.

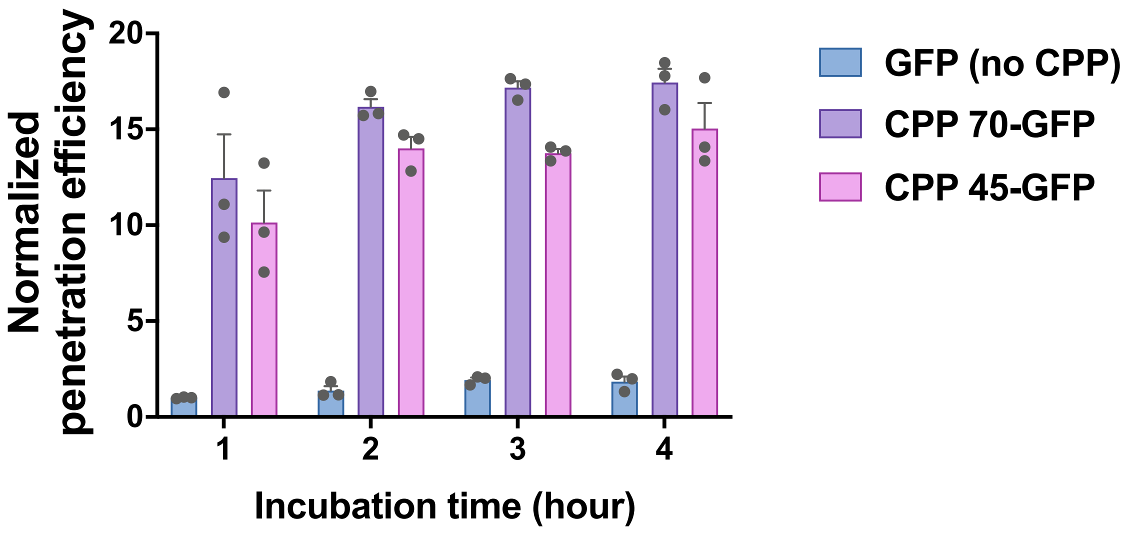

Supplementary Figure 4. Effects of CPP incubation time on GFP delivery efficiency

The GFP delivery efficiency of the two efficient CPPs, CPP 70 and CPP 45, was enhanced by increasing the incubation time after electroporation. The GFP fluorescence intensity of *E. coli* cells was saturated within 3 h of incubation time. The mean and standard error were calculated from three independent experiments.

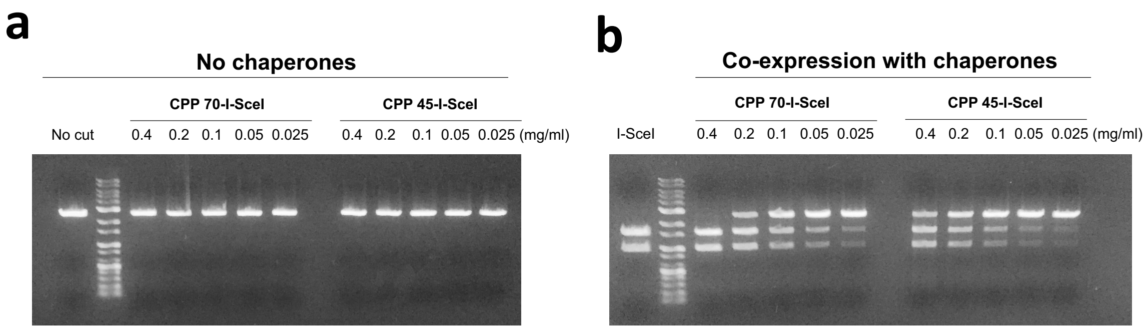

Supplementary Figure 5. Activity assay of CPP-conjugated I-SceI

a Two CPP-conjugated I-SceI proteins were expressed in *E. coli* BL21(DE3) without additional chaperones. The purified CPP-conjugated I-SceI proteins exhibited no activity. **b** The two proteins were expressed in *E. coli* BL21(DE3) with two additional chaperones (GroES and GroEL) from the pGro7 plasmid. The purified CPP-conjugated I-SceI proteins exhibited concentration-dependent activity.

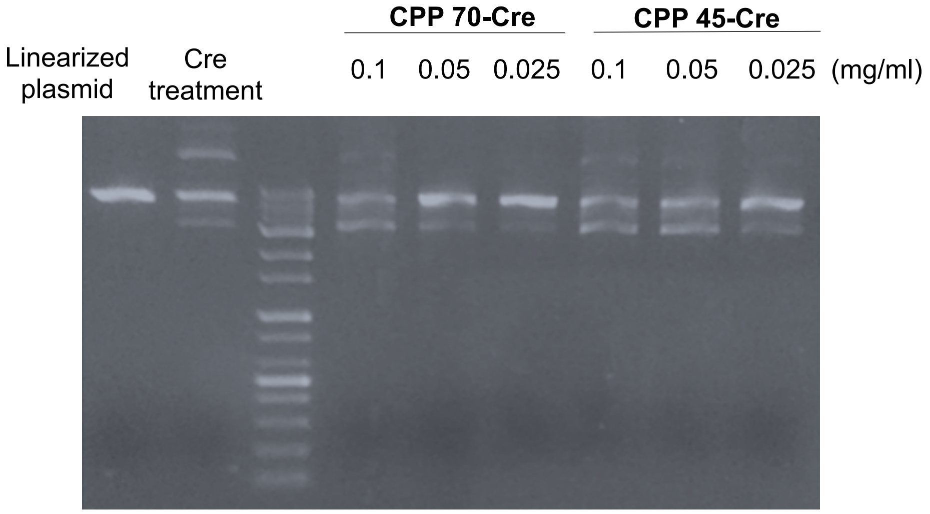

Supplementary Figure 6. Activity assay of CPP-conjugated Cre recombinase

Two CPP-conjugated Cre recombinases were expressed in *E. coli* BL21(DE3) in fusion with CBD and intein. The CPP-conjugated Cre recombinases were purified using CBD-intein tag, and the tag was removed from the proteins using thiol reagents such as dithiothreitol (DTT) according to the manufacture's instruction (New England BioLabs). The purified CPP-conjugated Cre recombinases excised the sequence flanked by loxP sites. The linearized plasmid structure is shown in Fig. 7c.

Supplementary Table 1. Determination of the optimal Ni^{2+} concentration for the selection of plasmid-removed *E. coli* cells

$NiCl_2$ concentration (mM)	Number of colonies	
	Wild-type cells	Plasmid-harboring cells
1.8	2492 ± 94.27	10.67 ± 2.27
1.9	2065.33 ± 181.07	4 ± 1.15
2	1010.67 ± 121.71	0.34 ± 0.19
2.1	776 ± 73.11	0
2.2	29 ± 4.40	0
2.3	0.67 ± 0.19	0
>2.30	0	0

Supplementary Table 2. Determination of the optimal Ni^{2+} concentration for the selection of marker gene-excised *Methylomoas* sp. DH-1 cells

$NiCl_2$ concentration (mM)	Number of colonies	
	Wild-type cells	*tetA*-harboring cells
0.5	180 ± 6.42	119 ± 6.42
1	128 ± 10.50	125 ± 4.40
2	114 ± 4.04	3 ± 2.08
4	87 ± 16.09	0
8	21 ± 5.03	0
>8.0	0	0
