# Supplementary material for: Identification of efficient prokaryotic cell-penetrating peptides with applications in bacterial biotechnology
Source: Commun Biol. 2021 Feb 15;4:205. doi: 10.1038/s42003-021-01726-w (PMC7884711; doi:10.1038/s42003-021-01726-w)
Supplement: Supplementary file 3 — Description of Additional Supplementary Files [file 42003_2021_1726_MOESM3_ESM.pdf]

Description of Additional Supplementary Files

File name: Supplementary Data 1

Description: List of CPPs used in this study.

File name: Supplementary Data 2

Description: All datasets used to draw main graphs in this study.
